# Supplementary material for: Ultrashort laser pulse doubling by metal-halide perovskite multiple quantum wells
Source: Nat Commun. 2020 Jul 17;11:3361. doi: 10.1038/s41467-020-17096-6 (PMC7368017; doi:10.1038/s41467-020-17096-6)
Supplement: Supplementary file 1 — Supplementary Information [file 41467_2020_17096_MOESM1_ESM.pdf]

Supplementary Information for

Ultrashort Laser Pulse Doubling by Metal-Halide Perovskite Multiple Quantum Wells

Jia Guo[1]†, Tanghao Liu[1]†, Mingjie Li[2]†*, Chao Liang[1], Kaiyang Wang[1], Guo Hong[1], Yuxin Tang[1], Guankui Long[3], Siu-Fung Yu[2], Tae-Woo Lee[4], Wei Huang[5], Guichuan Xing[1]*

[1]Joint Key Laboratory of the Ministry of Education, Institute of Applied Physics and Materials Engineering, University of Macau, Macao 999078, China.

[2]Department of Applied Physics, The Hong Kong Polytechnic University, Hung Hom, Kowloon, Hong Kong, P. R. China.

[3]School of Materials Science and Engineering, National Institute for Advanced Materials, Nankai University, Tianjin, 300350 P. R. China.

[4]Department of Materials Science and Engineering, Seoul National University (SNU), Seoul, Republic of Korea.

[5]Institute of Flexible Electronics (IFE), Northwestern Polytechnical University (NPU), Xi'an 710072, Shaanxi, P. R. China.

*Correspondence to: gcxing@um.edu.mo; ming-jie.li@polyu.edu.hk

†These authors contributed equally.

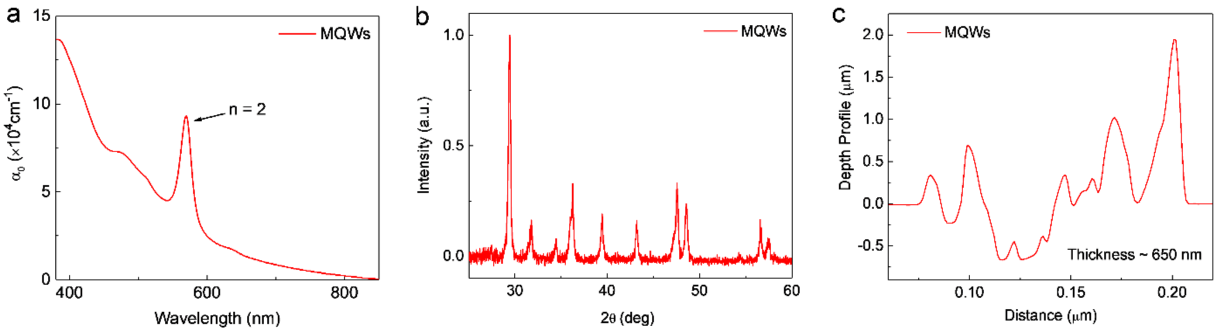

Supplementary Fig. 1 | Basic physical properties of the metal-halide perovskite multiple quantum wells (MQWs) film. a, UV-Vis absorption. **b**, X-ray diffraction (XRD). **c**, Step profile showing the thickness of the film.

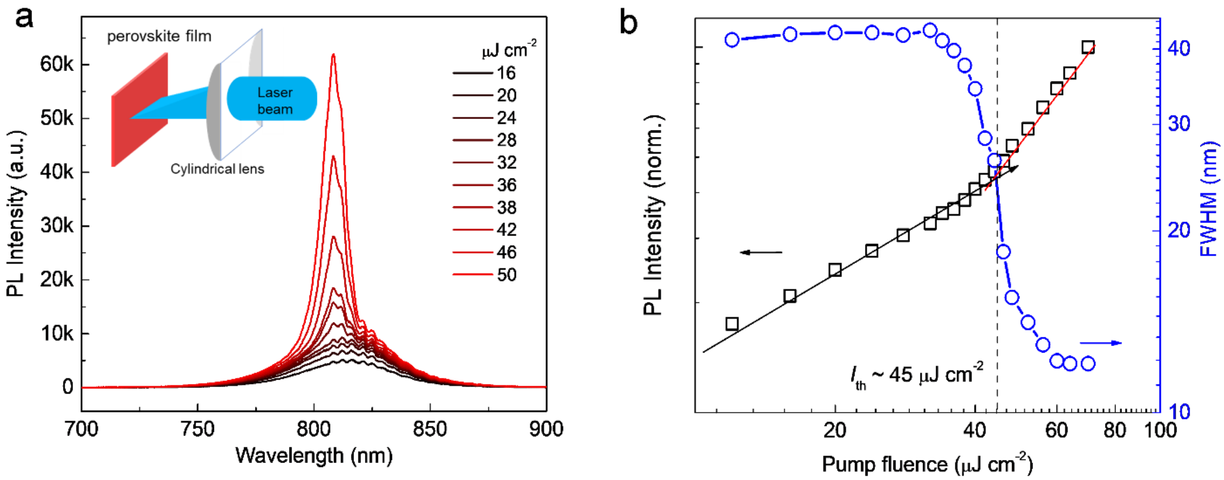

Supplementary Fig. 2 | Stimulated emission from 3D metal-halide perovskite ($FAPbI_3$) film. **a**, Photoluminescence (PL) spectra of 3D perovskite film under 400-nm fs-laser excitation with at different pump fluence. Inset shows the PL measurement configuration. **b**, Pump-fluence dependent PL intensities and the full width at half maximum (FWHM) of the emission bands.

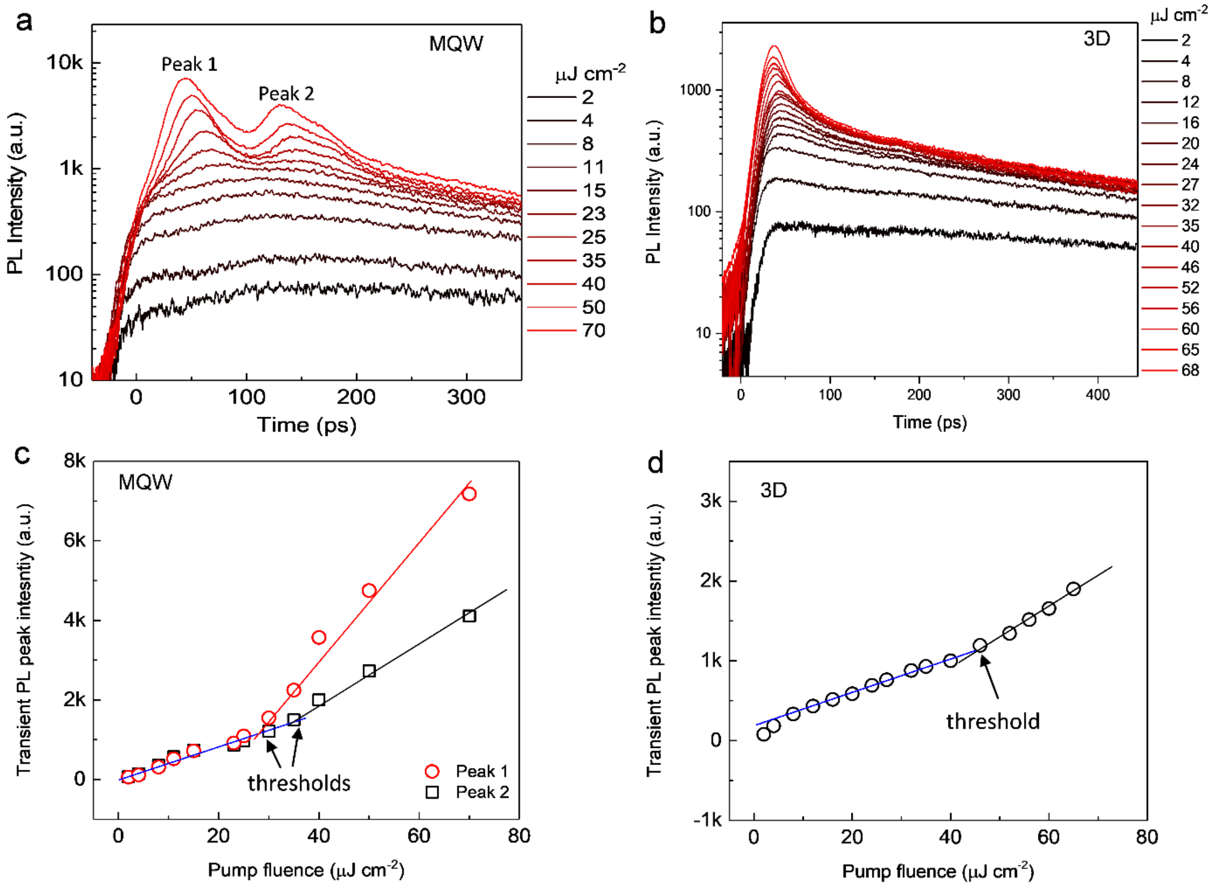

Supplementary Fig. 3 | Pump fluence dependent time-resolved photoluminescence (TRPL) of the perovskite films. a, TRPL decay curves of the perovskite multiple quantum wells (MQWs). **b**, TRPL decay curves of the 3D perovskite film. **c**, TRPL peak intensities as a function of pump fluence of the MQWs. The thresholds of stimulated emission (StE) for peak 1 and peak 2 are ~26 and 35 μJ cm^{-2}, respectively. **d**, TRPL peak intensities as a function of pump fluence of the 3D perovskite. The samples are excited by 400 nm, 50 fs, 1 kHz laser. The threshold pump fluence of transient TRPL intensity from peak 1 of MQW film and 3D perovskite film are consistent with measured StE threshold measured from time-integrated PL. The slightly higher threshold of transient peak 2 of MQW film may be due to the higher injection carrier density is needed for StE generation by the funneled carriers.

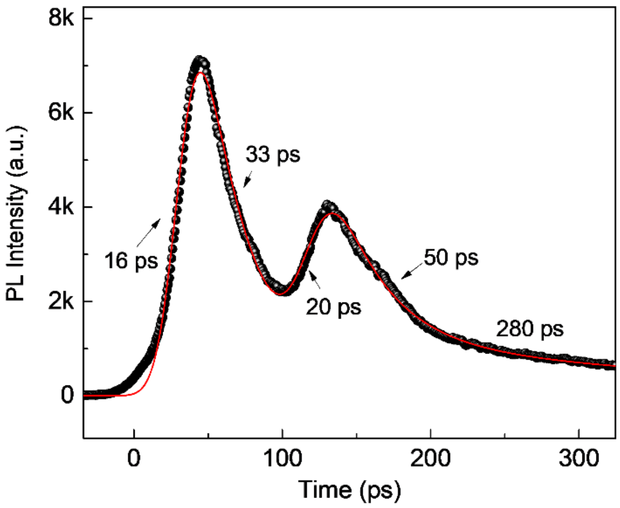

Supplementary Fig. 4 | Modelling the dual pulsed stimimulated emission from perovskite multiple quantum wells (MQWs). Time-resolved photoluminescence (TRPL) curve of the MQWs film excited with 400-nm laser pulse (50 fs, 1 kHz) under pump fluence of 70 μJ cm^{-2}. The red curve is the simulated result.

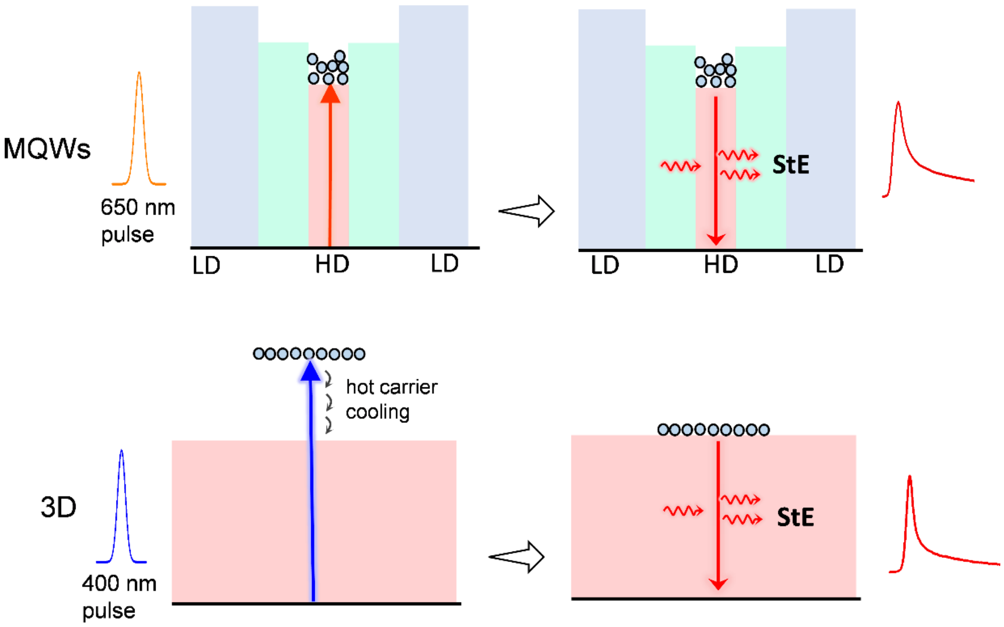

Supplementary Fig. 5 | Schematic carrier dynamics showing the single pulse stimulated emission. Schematic carrier dynamics of the carrier generation and stimulated emission processes in perovskite multiple quantum wells (MQWs) under 650 nm (50 fs, 1 kHz) laser pulse excitation and 3D perovskite under 400 nm (50 fs, 1 kHz) laser pulse excitation.

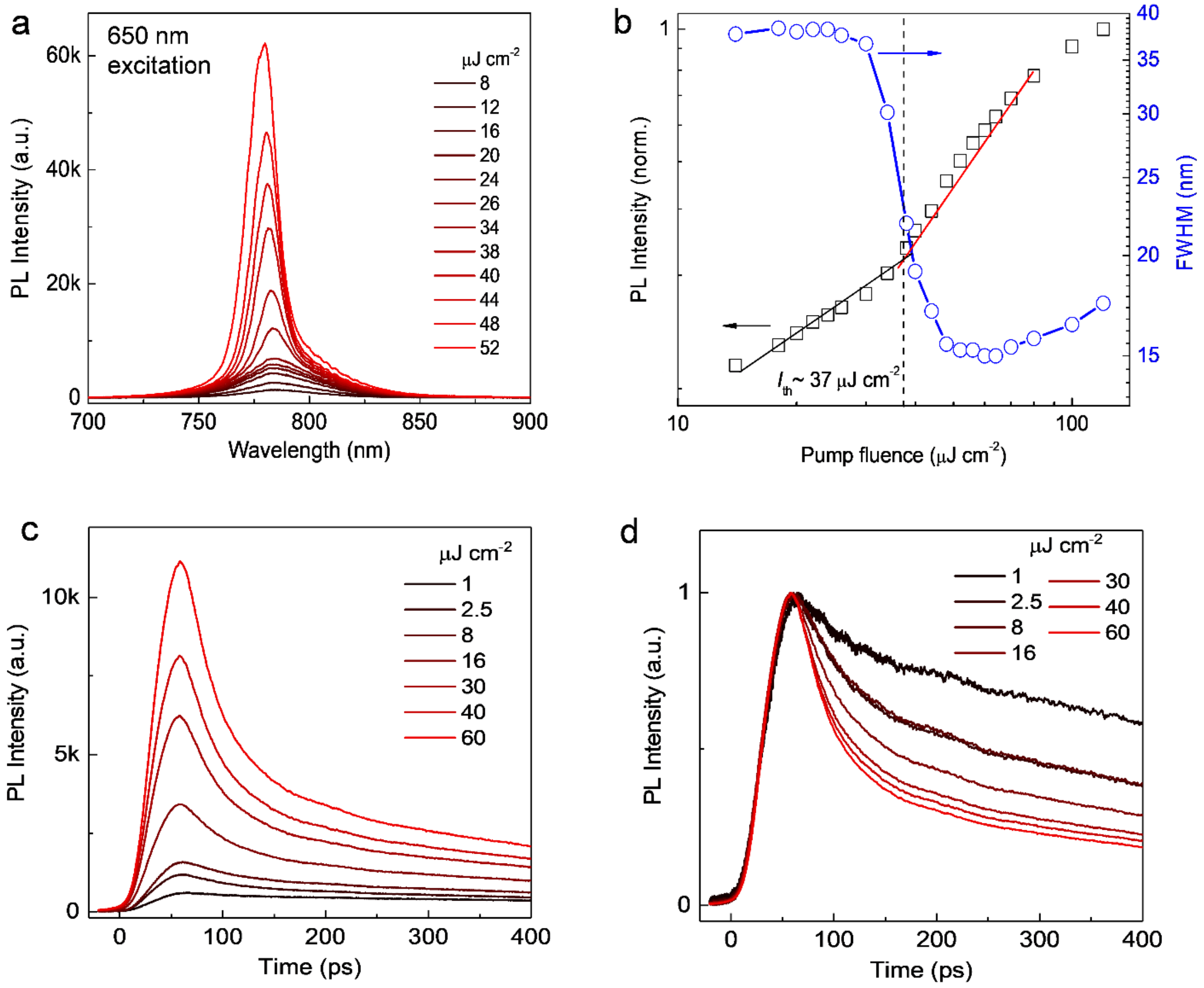

Supplementary Fig. 6 | Single pulse emission from the perovskite multiple quantum wells (MQWs) under 650 nm (50 fs, 1 kHz) laser pulse excitation. **a**, Photoluminescence (PL) spectra of MQWs under 650-nm fs-laser excitation at different pump fluence. **b**, Pump-fluence dependent PL intensities and full-width-at-half-maximums (FWHMs) of the emission bands. **c**, Pump-fluence dependent time-resolved PL (TRPL) decay curves. **d**, Normalized TRPL curves at different pump fluence.

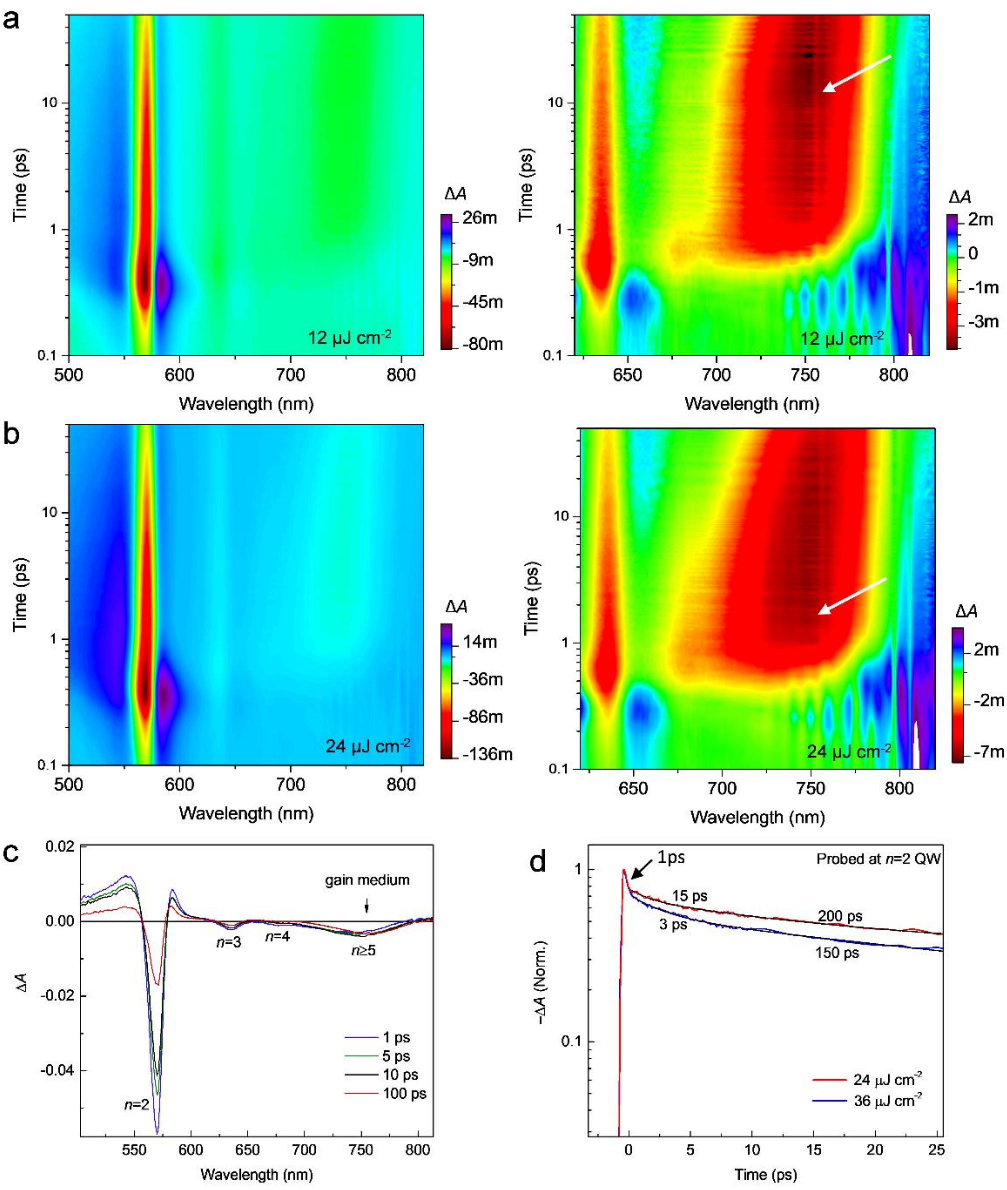

Supplementary Fig. 7 | Transient absorption (TA) of the perovskite multiple quantum wells (MQWs) film. 2D pseudo-color plot of TA spectra of the MQWs ($(NMA)_2(FA)_{n-1}PbnI_{3n+1}$) film under 400 nm (50 fs, 1 kHz) laser pulse excitation with pump fluence of **a**, 12 µJ cm^{-2} and **b**, 24 µJ cm^{-2}, respectively. The left and right panels of **a** and **b** are plots at different probe wavelength ranges. The white arrows indicate the peak positions of the TA bleaching intensity probed at the gain medium, which shows the shorter rise time of TA bleaching at higher pump fluence. **c**, Representative TA spectra at different delay time under pump fluence of 24 µJ cm^{-2}. The values of n in $(NMA)_2(FA)_{n-1}PbnI_{3n+1}$ listed in the figure correspond to the QWs with different inorganic layer numbers. The black arrow indicates the peak of the TA signal of gain media probed at the $n \geq 5$ QWs. **d**, Normalized TA dynamics probed at the $n = 2$ QWs with different pump fluences. The fitted lifetimes with bi-exponential decay function (black curve) are labeled. The fast decay at higher pump-fluence is consistent with the shorter building up of TA at $n \geq 5$ QWs.

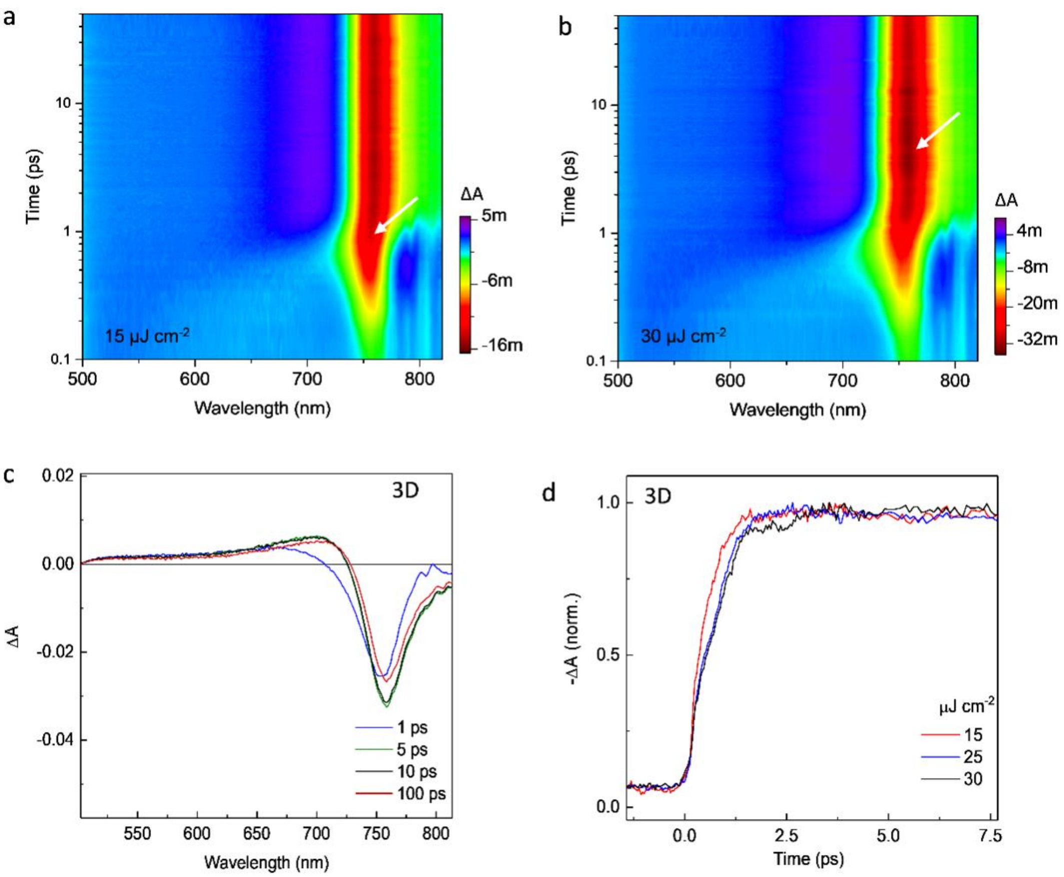

Supplementary Fig. 8 | Transient absorption (TA) of the 3D perovskite ($FAPbI_3$) film. 2D pseudo-color plot of representative TA spectra of 3D $FAPbI_3$ perovskite film under 400 nm (50 fs, 1 kHz) laser pulse excitation with pump fluence of **a**, 15 μJ cm^{-2} and **b**, 30 μJ cm^{-2}. The white arrows indicate the peak positions of the TA bleaching intensity, which show the delayed rise time of TA bleaching at higher pump fluence. **c**, Representative TA spectra at different delay time under pump fluence of 30 μJ cm^{-2}. **d**, Normalized TA building-up dynamics monitored at the bleaching peak of 3D perovskite film at different pump fluence.

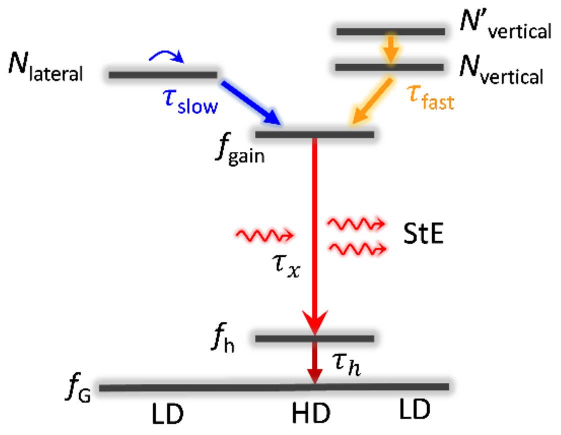

Supplementary Fig. 9 | Schematic energy level diagram showing the carrier funneling processes to the gain medium.

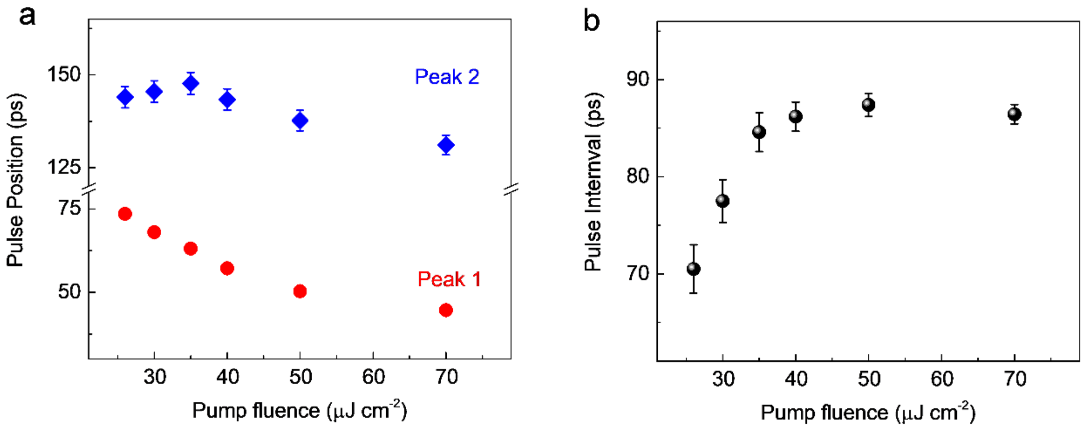

Supplementary Fig. 10 | Variation of the stimulated emission (StE) pulse intervals. **a** Pump-fluence dependent StE pulse positions. **b** Pump-fluence dependent intervals of two transient StE pulses. The error bars represent the uncertainties in the determination of StE pulse peak positions.

Supplementary Table 1. The parameter values used in the above equations to numerically calculate the $I_{PL}(t)$ curves.

τ_{slow} (ps)	25	21	17	13	10	5	2.5
R	3	4	4.5	5.8	6.5	8.6	10
x	0.6	0.63	0.66	0.66	0.68	0.75	0.75

Supplementary Note 1 | Carrier funneling model

We use a phenomenological numerical model to describe the carrier relaxation and recombination dynamics in the mixed-dimensional perovskite MQWs system. Supplementary Fig. 9 shows the energy diagram of the inter-level relaxation and recombination processes in the MQW system. The charge carriers are first generated in the dominant bilayer QWs by 400-nm laser pulse. The injected electrons have two channels to transfer to the gain excited state level, which can recombine with holes in the valence band to generate the PL. Because the large lateral to vertical dimension ratio (aspect ratio) of LD QWs, we assume that the lateral charge transfer from the near LD QWs (dominant n = 2 perovskite QWs) to HD QWs ($n \geq 5$ QWs) would be mainly the process in the QW with the relatively slow transfer time (τ_{slow}). And the vertical charge transfer would be the charge transfer from the far LD QWs across the stacked QWs with the fast transfer time (τ_{fast}) via quantum coupling. The probability of lateral charge transfer could be proportional to the charge occupation probability and state density in the LD QWs and the charge non-occupation probability of HD QWs; whereas the vertical charge transfer could be proportional to the state density in the LD QWs not connected with HD QWs (N'_{vertial}), the charge non-occupation probability of the LD QWs connected with HD QWs (N_{vertial}) and the charge non-occupation probability of HD QWs. The emission of gain medium is defined as the recombination of the transferred electrons in the excited states of gain medium with the unoccupied states in hole states. We then formulate the following coupled rate equations to describe the emission dynamics of gain medium as represented by $I_{\mathrm{PL}}(t)$:

$$N_{\mathrm{G}} \frac{df_{\mathrm{G}}}{dt} = N_h \frac{f_{\mathrm{h}}(1-f_{\mathrm{G}})}{\tau_h} \quad (1)$$

$$N_{\mathrm{h}} \frac{df_{\mathrm{h}}}{dt} = N_{\mathrm{gain}} \frac{f_{\mathrm{gain}}(1-f_{\mathrm{h}})}{\tau_x} - N_h \frac{f_{\mathrm{h}}(1-f_{\mathrm{G}})}{\tau_h} \quad (2)$$

$$N_{\text{gain}}\frac{df_{\text{gain}}}{dt} = -N_{\text{gain}}f_{\text{gain}}(1-f_{\text{h}})/\tau_x + N_{\text{lateral}}\frac{f_{\text{lateral}}(1-f_{\text{gain}})}{\tau_{\text{slow}}} + N'_{\text{vertial}}(1-f_{\text{vertial}})\frac{(1-f_{\text{gain}})}{\tau_{\text{fast}}} \tag{3}$$

$$N_{\text{lateral}}\frac{df_{\text{lateral}}}{dt} = -N_{\text{lateral}}\frac{f_{\text{lateral}}(1-f_{\text{gain}})}{\tau_{\text{slow}}} \tag{4}$$

$$N_{\text{vertial}}\frac{df_{\text{vertical}}}{dt} = -N'_{\text{vertial}}(1-f_{\text{vertial}})\frac{(1-f_{\text{gain}})}{\tau_{\text{Fast}}} \tag{5}$$

$$1/\tau_x = \left(1 + Re^{f_{\text{gain}}-f_{\text{h}}}\right)/\tau_{x0} \tag{6}$$

$$I_{\text{PL}}(t) \sim f_{\text{gain}}(1-f_{\text{h}})/\tau_x \tag{7}$$

In the above equations, f_G and f_h represent the ground and valence band hole occupation probabilities, f_{gain}, $f_{lateral}$ and $f_{vertical}$ represent the conduction band electron occupation probabilities in the gain medium, the LD bilayer QWs related with lateral and vertical carrier funneling processes to the gain medium, respectively. *N* represents the state densities at the different energy levels. τ_{slow} and τ_{fast} are the slow and fast carrier funneling lifetime to the gain excited state via lateral charge transfer in the bilayer QW and vertical charge transfer across the bilayer QWs, respectively. *R* represents the recombination strength at different excitation levels of *x*. Initial conditions: f_{G}=1-*x*; f_{h}= 0; f_{gain}= 0; f_{lateral} = *x*; f_{vertial} = 0; τ_{x0} = 5 ns; τ_h =2 ps. The values of variables used in the simulated I_{PL}(t) shown in Fig. 3b in the main text are listed in the Supplementary table 1.
